# The association between the number of office visits and the control of cardiovascular risk factors in Iranian patients with type2 diabetes

**DOI:** 10.1371/journal.pone.0179190

**Published:** 2017-06-30

**Authors:** Sedighe Moradi, Zeinab Sahebi, Ameneh Ebrahim Valojerdi, Farzaneh Rohani, Hooman Ebrahimi

**Affiliations:** 1 Endocrine research center, Institute of endocrinology and metabolism, Iran University of Medical Sciences, Tehran, Iran; 2 Endocrine research center, Institute of endocrinology and metabolism, Division of biostatistics, Iran University of Medical Sciences, Tehran, Iran; 3 Endocrine research center, Institute of endocrinology and metabolism, Research Center for Growth & development in Childhood and adolescence, Iran University of Medical Sciences, Tehran, Iran; 4 Students’ scientific research center, Tehran University of Medical Sciences, Tehran, Iran; Universita degli Studi di Perugia, ITALY

## Abstract

**Introduction:**

Patients with diabetes type2 should receive regular medical care. We aimed at investigating the association between the number of office visits and improvement of their cardiovascular-risk factors.

**Methods:**

Four hundred and ninety patients with type 2 diabetes mellitus who were followed in a tertiary center were enrolled in this longitudinal study. The minimum follow up period was 3 years. Patient data were extracted from manual or electronic records.

**Results:**

Sixty- four percent of cases were females, the mean age was 61 ± 12.45 years, and the mean disease duration was 6.5 ±7.9 years. The mean number of office visits was 2.69 ± 0.91 per year. Comparing the means of each of the cardio-vascular risk factors showed a significant decrease in all cardiovascular risk factors, while there was a significant weight gain over the same period. The association between changes in these parameters and the number of patients’ office visits per year were not statistically significant. In patients with disease duration less than 5 years, each additional office visits by one visit per year was associated with a decrease in serum total cholesterol by 6.94 mg/dl. The mean number of office visits per year in patients older than 60 years old was more than younger patient (p = 0.001).

**Conclusion:**

The decrease in the mean values of the investigated parameters was statistically significant between the first year of follow up and the following years. Yet, these changes were not related to the mean number of patients’ office visits per year, which may reflect the poor compliance of patients to treatment regardless of the number of their office visits.

## Introduction

Diabetes mellitus is the most prevalent metabolic disorder, and a serious disease with a large financial burden [1]. Diabetes can be associated with several long-term complications that increase mortality and morbidity and affect the quality of life negatively [2].

Regular medical care can prevent many of the common complications of diabetes [3]. According to the American Diabetes Association (ADA) recommendations, checking the glycosylated hemoglobin (HbA1c) every three months aids in setting a glycemic target for the patient that needs to be achieved and maintained. Yet, the frequency with which HbA1c should be checked depends on the clinical status of the patient, the treatment regimen, and the physician’s judgment. For most patients with stable and within-target glycemia, doing the test twice per year may be enough, while in patients with unstable blood glucose or those requiring a more strict control, HbA1c may need to be checked even more frequently than once every three months [4].

Despite the important effect of preventive care in diabetes, many patients are not followed adequately as per the recommendations. One contributing factor is that these patients simply do not have the recommended regular office visits. Furthermore, many patients with diabetes have other concurrent chronic diseases, which may confuse the physician in terms of implementing the adequate follow up recommendations. Some studies show that patients who have more frequent office visits have a better outcome [5]. Recent guidelines do not include recommendations regarding the number and frequency of office visits by patients [6]. The set time intervals for applying therapeutic adjustments and performing laboratory tests range from 3 days for insulin dosage adjustment to 3 months for checking HbA1c, yet the benefits of repeated office visits may not be limited to adequate therapy or performing laboratory tests [6, 7]. In a study, patients who had an office visit every 1–2 week needed less average time to achieve an HbA1C<7% and target blood pressure and LDL, compared to those who had office visits every 3–6 months [5]. Another study showed that, over the 3-years study period, patients who had 3–4 office visits per year had a lower mean HbA1c compared to those with 1–2 visits per year [8]. More than one third of the American patients with diabetes have less than 4 office visits per year [9]. From all physician visits in 2010, 11% of them were attributed to diabetic patients. In 87% of clinic visits, medications were either prescribed or continued and the number of medications increased with age as well [10].

Based on the fact that diabetes has a high prevalence and burden, studies that can improve diabetes control and prevent its complications, are of utmost importance. Thus, in this study, we aimed to investigate the effect of the number of physician visits on the control of blood sugar and other related parameters.

## Material and methods

This is a longitudinal study conducted on patients with type 2 diabetes mellitus. Data of patients with type 2 diabetes (as per ADA criteria), who were followed in the diabetes clinics of The Institute of Endocrinology and Metabolism-Firoozgar Hospital between 2006 and 2011, were extracted from the manual or electronic records and were then documented in preset questionnaires. All patients had been followed by endocrinologist for a minimum period of 3 years. Before starting the *visit*, an informed consent form had to be *filled* in by the participants and the research was carried out in compliance with the *Helsinki Declaration*. The study was approved by ethic committee of Iran University of Medical Sciences (IUMS). Demographic, medical, and laboratory data of patients including age, gender, level of education, duration of diabetes, blood pressure, physical activity, diet, smoking status, medications, micro and macrovascular complications, comorbidities, the number of office visits, and results of laboratory tests fasting (FBS), 2 hours post prandial (2hpp BS), HbA1c, serum cholesterol, LDL, HDL, TG, BUN, creatinine, albuminuria) were collected at each visit and entered into the SPSS system as a mean value every three months. The minimum sample size was estimated to be 400 patients, yet due to deficiency in some data we had to include more patients. Patients with a follow up period of less than 3 years, those with deficient data, and cases followed by non-specialist doctors were excluded from this study.

### Statistical analysis

Analyses were performed using the SPSS-19 software. Excel 2010 was used for some calculations. First, We used histograms for investigating normal distributions, then based on whether the variables had a normal distributions or not, results were reported as mean and standard deviation or median and range. On this basis, we used a paired t-test.

With the help of statistic description indices, output data were reported as mean ±standard deviation or as frequency and percentage. The differences between the mean values of the investigated parameters in the first year and those performed in the following years were analyzed. T-test, chi-square, and univariate and multivariate linear regression models were used in data analysis. The scatter plot was used to investigate relationships between quantitative variables.

## Results

This study included 498 patients with type 2 diabetes with a minimum follow up period of three years. Among these, 279 patients had a follow up of more than four years. Patient characteristics are shown in “Table 1” “S1 Dataset”. In the study population 35.5% were male, and 64.3% were female, with a mean age of 61± 12.45 years and mean disease duration of 6.5 ±7.9 years.

**Table 1:** Basal characteristics of patients.

Characteristics	Mean/frequency	Number*
**Mean age (year)**	61 ±12.45	491
**Minimum age**	20	
**Maximum age**	92	
**Male**	177 (35.5%)	497
**Female**	320 (64.3%)	
**Diabetes duration (year)**	6.5 ±7.9	483
**Minimum (month)**	1	
**Maximum (year)**	33	
**Smoking%**	69 (14%)	479
**BMI (Kg/m**^**2**^**)**	28.5 ± 5.03	484
**Hypertension**	336 (82%)	410
**Hyperlipidemia**	404 (91.4%)	442
**CVD positive**	91 (27.3%)	333
**Retinopathy**	38 (30.8%)	118
**Neuropathy**	128 (51.8%)	247
**Nephropathy**	37 (17.35%)	213
**Education level**		
**Lower than diploma**	284 (57%)	
**Diploma**	126 (25.3%)	
**Bachelor**	59 (11.8%)	
**Higher than bachelor**	2 (4%)	

BMI = Body mass index, CVD = Cardio vascular disease.

* Total number of patients for whom data were valid

There was a significant decrease in the mean diastolic blood pressure, fasting blood sugar, 2hpp glucose, triglyceride, total cholesterol, and LDL between the first year and the following years of follow up “Table 2”. At the same time there was a significant increase in the mean weight of the patients “S1 Dataset”.

**Table 2:** The mean values for the investigated variables in the first year compared to the following years.

Variable	First year mean (±SD)	Follow-up years mean (±SD)	Total	P-value
**SBP (mm/Hg)**	128.87±14.16	127.75±13.14	488	0.055
**DBP (mm/Hg)**	81.60±6.01	79.77±5.40	488	0.000
**FBS (mg/dl)**	160.08±56.04	146±36	475	0.000
**2hppG (mg/dl)**	223.71±82.48	205.41±60.57	415	0.000
**HbA1c (%)**	7.34±1.78	7.55±1.19	336	0.037
**Cholesterol (mg/dl)**	174.95±39	160.86±29.54	418	0.000
**LDL (mg/dl)**	96.49±29.70	87.62±26.82	396	0.000
**TG (mg/dl)**	161.89±84.69	149.89±64.33	428	0.000
**HDL (mg/dl)**	45.15±10.15	45.64±9.35	384	0.344
**Cr (mg/dl)**	1.01±0.55	1.02±0.33	345	0.885
**Albuminuria (mg/dl)**	69.25±10.92	66.36±8.2	183	0.777
**Weight (Kg)**	72.57±13.07	74.36±13.20	487	0.000

SBP: systolic blood pressure, DBP: diastolic blood pressure, FBS: fasting blood sugar, 2hppG: 2-hour postprandial glucose, HbA1c: glycosylated hemoglobin, LDL: low-density lipoprotein, HDL: high-density lipoprotein, TG: triglyceride, Cr: serum creatinine.

Regarding treatment, at the beginning of the study 134 patients were on insulin therapy whereas at the end this number increased to 207 patients and this increase was statistically significant (p<0.001). In this study patients had a mean of 2.6 office visits per year (range: 0.5–7.5). The number of patients who achieved the ADA 2015 goal criteria for each of the investigated parameters (blood pressure, LDL, HbA1c, FBS, 2hpp glucose) is shown in “Table 3” “S1 Dataset”.

**Table 3:** The number of patients who achieved the ADA 2015 goal for each of the investigated parameters (blood pressure, LDL, HbA1c, FBS, 2hpp glucose).

Variable	Frequency (%)	Total
**Sys BP<140(mm/Hg)**	402(81.4)	494
**DBP <90(mm/Hg)**	465(93.6)	497
**BP<140/90(mm/Hg)**	395(80)	494
**LDL<70(mg/dl)**	167(33.9)	493
**FBS<130(mg/dl)**	205(41.3)	496
**2hpp<180(mg/dl)**	189(38.3)	493
**HbA1c <7%**	200(41.8)	479
*All parameters	55(11.5)	475

SBP: systolic blood pressure, DBP: diastolic blood pressure, BP: Blood pressure, LDL: low density lipoprotein, FBS: fasting blood sugar, 2hppG: 2-hour postprandial glucose, HbA1c: glycosylated hemoglobin.

*Patients who achieved goal criteria for all cardio-vascular risk factors

Linear regression analysis was performed to evaluate for a possible relation between the investigated variables and the “mean number of office visits” of patients per year as an independent variable “Tables 4, 5 and 6” “S1 Dataset”.

**Table 4:** Binary logistic regression between goal outcomes* and mean number of visit per year, adjusted by sex and age.

Variable	P-value	OR (95%CI)
**FBS(mg/dl)**	0.59	1.06(0.87–1.29)
**2hppG(mg/dl)**	0.64	0.95(0.78–1.17)
**HbA1c(mg/dl)**	0.99	0.99(0.81–1.23)
**LDL(mg/dl)**	0.32	0.900(0.73–1.11)
**BP(mm/Hg)**	0.24	0.86(0.68–1.10)

CI: confidence interval, FBS: fasting blood sugar, 2hppG: 2-hour postprandial glucose, HbA1c: glycosylated hemoglobin, BP: blood pressure, LDL: low-density lipoprotein.

*Correlation between achieving optimal cardiovascular risk factor control as defined by ADA (4) with mean number of visit per year

**Table 5:** Linear relationship between changes in cardiovascular risk factors and mean number of visit per year.

Dependent variable	B±SE	Standardized B	P—value
**Change FBS**	0.45±2.68	0.008	0.86
**Change 2hpp**	7.50±4.06	0.090	0.06
**Change HBA1c**	-0.10±0.11	-0.049	0.37
**Change SYS BP**	0.26±0.64	0.018	0.68
**Change DBP**	-0.15±0.27	-0.025	0.57
**Change TG**	2.60±3.58	0.035	0.46
**Change TC**	0.54±1.96	0.014	0.78
**Change LDL**	-2.34±1.66	-0.071	0.15
**Change HDL**	0.26±0.57	0.024	0.46
**Change Weight**	-0.08±0.21	-0.017	0.70

FBS: fasting blood sugar, 2hppG: 2-hour postprandial glucose, HbA1c: glycosylated hemoglobin, SBP: systolic blood pressure, DBP: diastolic blood pressure, TG: triglyceride, TC: Total cholesterol, LDL: low density lipoprotein, HDL: high density lipoprotein,

B: regression coefficient

**Table 6:** Linear relationship between mean of individual's cardiovascular risk factors and mean number of visit per year.

Dependent variable	B±SE	Standardized B	P—value
**Mean FBS**	-1.13±1.68	-0.028	0.50
**Mean 2hpp**	-5.62±2.89	-.0082	**0.052**
**Mean HBA1C**	-0.008±0.07	-0.005	0.91
**Mean SYS BP**	-0.04±0.54	-0.003	0.94
**Mean DBP**	0.15±0.22	0.026	0.50
**Mean TC**	-1.99±1.41	-0.061	0.16
**Mean TG**	-2.46±2.68	-0.035	0.36
**Mean LDL**	0.10±1.34	0.003	0.94
**Mean HDL**	0.21±0.47	0.020	0.65
**Mean Weight**	0.95±0.01	0.004	0.76

FBS: fasting blood sugar, 2hppG: 2-hour postprandial glucose, HbA1c: glycosylated hemoglobin, SBP systolic blood pressure, DBP: diastolic blood pressure, TG: triglyceride, TC: Total cholesterol, LDL: low density lipoprotein, HDL: high density lipoprotein,

B: regression coefficient

**Fig 1:**
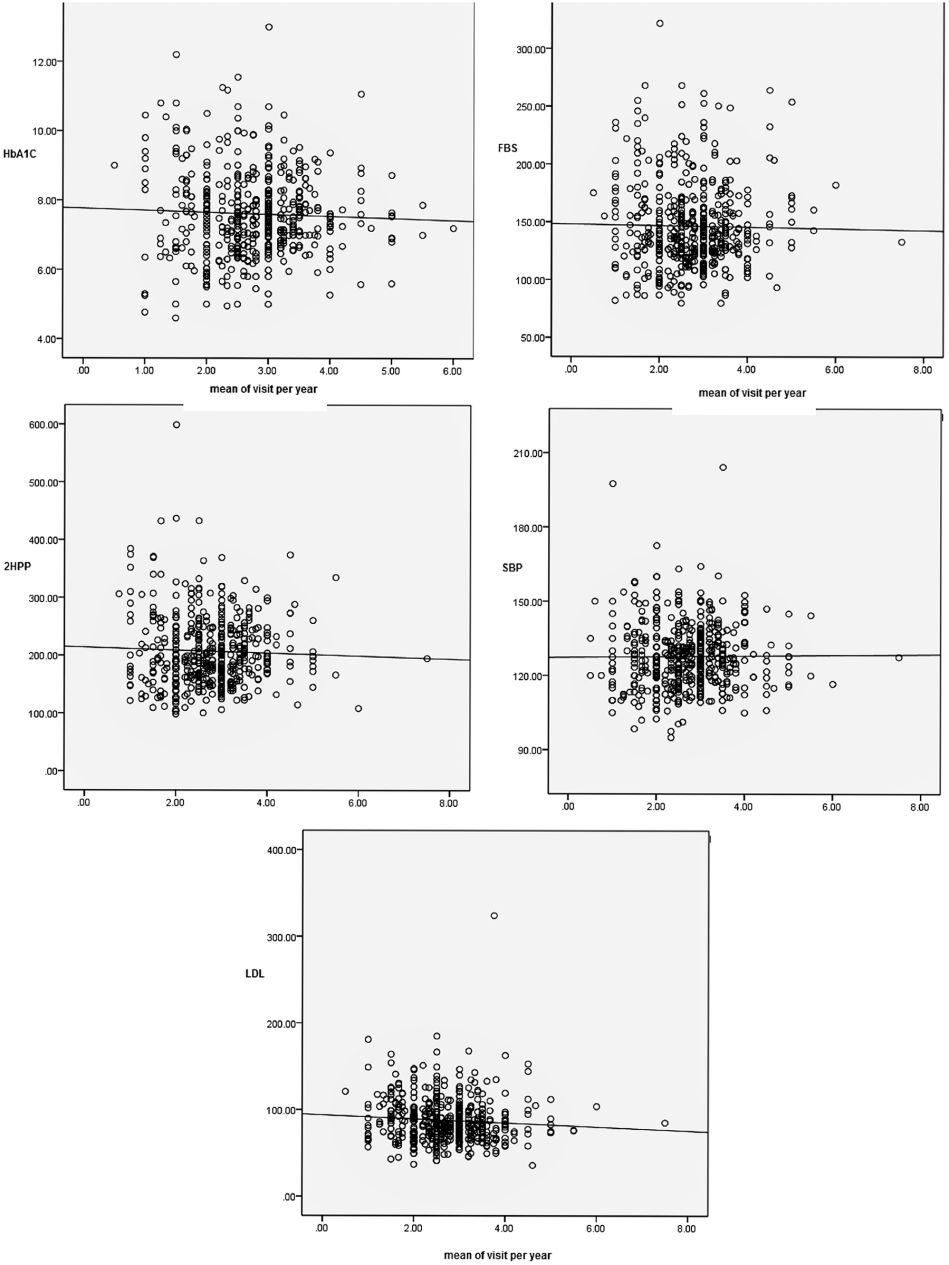
The relation between the mean values of the investigated variables and the mean number of office visits per year using a scatter plot. The lines in Fig 1 are ordinary least squares (OLS) bivariate regression lines.

From a statistical viewpoint there was no association between the investigated variables and the mean number of patients’ office visits per year. “Fig 1” shows the relation between the mean values of the investigated variables and the mean number of office visits of patients per year using a scatter plot “S1 Dataset”.

Furthermore, we did not find any significant relation between changes in the investigated variables and the number of office visits in the subgroup analysis of patients with ≤3 office visits per year as compare to those with ≥3 visits per year.

Linear regression analysis was performed for those patients with disease duration of less than 5 years (213 patients). In this analysis, decrease in the 2-hour post-prandial blood glucose was significantly correlated with the number of patients’ office visits (P = 0.004). In addition, according to the results, in this group of patients an additional one visit per year was associated with a decrease in mean cholesterol by 6.94 mg/dl (P = 0.041).

In subgroup analysis the percentage of males who achieved the LDL goal of≤70mg/dl were higher than female (39.7% VS 30.5%; P = 0.04), but regarding blood pressure control female achieved better control (86% VS 73%; P = 0.001), which were both statistically significant. Moreover, a subgroup analysis was performed to compare the investigated variables in individuals below and above 60 years of age at the end of the study. Individuals above 60 years of age had a significantly higher mean number of office visits per year (2.8±0.9 VS 2.56±0.88; P = 0.001), and also a significantly higher percentage of controlled postprandial serum glucose (P = 0.009).

## Discussion

We investigated the effect of the number of office visits per year on the control of blood sugar and the other cardiovascular risk factors. In our study, patients had an average of 2.6 visits per year. Regarding the ADA 2015 guidelines which recommend the measurement of HbA1c every 3 months, the mean values patient visits in this study were below the recommended level [4]. Considering the irregular time intervals between office visits it seems that these visits were not pre-planned. Comparing the means of the investigated variables at the beginning of the study and the following years, there was a significant decrease in the mean values of the variables, including fasting blood glucose (FBS), 2-hour postprandial (2hpp) glucose, diastolic blood pressure (DBP), serum cholesterol, triglyceride (TG), and low density lipoprotein (LDL), and a significant increase in the mean weight. Logically, this reduction could be the effect of medical interaction between patients and the physician. However, investigating the relation between the means of these variables and the mean number of patients’ office visits per year; we found no significant correlation. These results reflect an improvement in the control of the relevant parameters after the first year of follow up, yet, this improvement was not an effect of the number of patients’ office visits per year. The way an office visit is performed and patient adherence are of utmost importance in this regard, and since diabetes management is a multifactorial issue that requires cooperation between the patient and the physician, non-adherent patients have less benefit from office visits. A good glycemic control is based on effective self-management by the patient. Thus, structured education about life style modification, weight reduction and adherence to medications is fundamental for diabetes management. According to the study results, it seems that may be’ visits to the clinic were limited to prescription of medications.

In order to achieve more accurate results, we performed further subgroup analysis, whereby LDL was the only variable that had more reduction with the increase in the number of office visits. This may be due to the simpler guidelines concerning the use of statins and the easier administration of these medications (once daily) in comparison to the anti-diabetic and anti-hypertensive medications. Another reason may be that LDL is less affected by lifestyle (nutrition and physical activity) than serum glucose and requires less education.

In a study conducted in Canada, the authors divided barriers to optimizing glycemic control into 3 groups as follows: barriers related to the patient, clinic, and system, respectively. Among all the barriers, those related to the patients were most prominent [11].

Some studies showed that patients who had more physicians’ visits tended to have better outcomes. In a previous study, patients who had a clinic visit every 1 to 2 weeks needed less average time to achieve an HbA1c level of <7% than patients who had a clinic visit every 3 to 6 months. Visits once every 2 weeks were associated with a more rapid achievement of target HbA1c and low-density lipoprotein (LDL) levels [6]. Likewise, in another retrospective cohort study, patients with time intervals of <1 month between visits attained a normal blood pressure after 1.5 months, shorter than the 2.12 months in patients with intervals between visits exceeding 1 month [12]. In another study, patients with 3 or 4 visits per year had lower mean HbA1c levels over the 3-year study period than those with 1 or 2 visits per year [8]. One difference between our study and the other studies is that we did not investigate the time needed to achieve control and the intervals between visits, knowing that short intervals of, for example, 2 weeks are uncommon in our clinics. Moreover, patient adherence to treatment, which is an important factor in the control of diabetes, was not investigated. Studies show that more than half of patients with diabetes are not compliant to oral medications and insulin, and that non-adherence is consistently associated with poor glycemic control, where HbA1c level was 0.4–0.7% higher in non-adherent than in adherent patients. In one study, forgetfulness was an important cause of non-adherence to medications [13]. This issue could be more prominent with diabetes and hypertension than with hypercholesterolemia because of the short half-life of medications and the need for multiple daily doses.

Despite the improvement in glycemic control in adults, <15% of patients with diabetes achieve the target serum glucose level, blood pressure, and LDL level simultaneously [14, 15]. Studies showed that about 50% of patients with diabetes failed to achieve the target glycemic control despite having access to adequate treatment, and the physician’s failure to escalate treatment during the pre-planned visits is an important factor in this regard [16]. In our study, about 80%, 41%, and 38% of the patients achieved the target blood pressure, fasting blood glucose level, and 2-h postprandial glucose level, respectively. An LDL level of <70 mg/dL was reached in 41% of the cases, and an HbA1c level of <7% was achieved in 41% of cases as well. Achieving the target values for all the investigated parameters simultaneously was observed in 11.5% of the patients. It is noteworthy in this regard that our target LDL level was considered 70 mg/dL instead of 100 mg/dL and that our patients had a higher mean age, which means that they had a higher target HbA1c level according to the current guidelines. Moreover, elderly people tend to have less ability concerning self-care.

Considering these results, the differences in achieving the target values for the different parameters were comparable with the results of other studies. Data from the Center for Disease Control and Prevention show that between 2007 and 2009, 26% of patients with type 2 diabetes were receiving insulin therapy [17]. In our study, initially 26.9% of the patients were receiving insulin treatment, which increased to 41.5% at the end of the study. This means that 63 patients started receiving insulin from their physicians during the study period. We divided our patients into 2 age groups as follows: <60 and >60 years of age. The mean number of visits per year was 2.5 for patients aged <60 years and 2.8 for those aged >60 years. The target postprandial glucose level was achieved in 45% of the patients aged <60 years and in 33% of those aged >60 years, indicating a statistically significant difference. The treatment of elderly patients with diabetes becomes more complex with the simultaneous presence of other chronic diseases such as cognitive disorders, depression, and physical incapacity, in addition to the higher number of medications taken by these patients [18–21]. Therefore, when elderly patients make office visits every 3 to 6 months, the partial therapeutic changes applied during these visits are often insufficient for achieving glycemic control [22]. In a clinical trial that investigated the barriers to optimizing the management of elderly patients with diabetes, the most frequently encountered factor was insufficient treatment, which was initially due to patients’ desire to avoid changing the insulin dosage during the intervals between office visits or at times of depressed mood [23]. Given the above-mentioned points, the mean age of the patients in our study was relatively high. According to the World Health Organization report, patients with chronic diseases have a rate of adherence to treatment of approximately 50%. In developed countries, the rate of adherence is lower for patients with chronic diseases than for those with acute diseases, as compliance decreases significantly after the first 6 months [24, 25]. These factors could impede the self-management of patients with diabetes [25]. In this study, we made a comparison between men and women concerning the achievement of control targets. The results showed that the men achieved the target LDL values more frequently than the women, but a higher percentage of women achieved the target blood pressure. For further investigation, we performed a subgroup analysis between patients with ≤3 office visits per year and those with >3 visits per year, whereby the only significant difference was related to the decrease in mean weight, which was significantly higher in the patients with >3 visits per year. Considering the mean disease duration, which was 6.5 **±**7.9 years, a subgroup analysis performed for patients with a disease duration of <5 years revealed that an increase in the mean number of yearly office visits by one visit was associated with a decrease in the mean cholesterol level by 6.94 mg/dl.

As for the limitations of this study, first, it was a retrospective study and cannot be used to analyze all the patients’ behaviors during this period. Another limitation was that we could not assess the main barriers to optimizing glycemic control, such as compliance to treatment and adherence to nutrition therapy and physical activity recommendations. Furthermore, owing to the irregularly spaced office visits, the time intervals between these visits could not be assessed.

One of the strengths of this study was the adequate follow-up period and that the patients were in almost similar conditions regarding the clinics, healthcare providers, and health-care system, and were all followed by an endocrinologist.

In conclusion, this study investigated the association of patients’ office visits with improvement in disease-related parameters in patients with type 2 diabetes. Significant decreases in the mean values of the cardiovascular risk factors were found between the first year of follow-up and the following years. However, these changes had no significant relationship with the patients’ mean number of office visits per year. We suggest organizing educational classes parallel to the clinics and ensuring adequate patient involvement in such activities through electronic alerts and family members, in addition to the implementation of simpler and better-tolerated therapeutic regimens and creating a motivation for the patient through psychological counseling.

## Supporting information

S1 Dataset “The minimal dataset file”. (SAV)

## References

[1] Wild S, Roglic G, Green A, Sicree R, King H. Global prevalence of diabetes: estimates for the year 2000 and projections for 2030. Diabetes care. 2004;27(5):1047–53. 15111519 10.2337/diacare.27.5.1047

[2] Martin S, Schneider B, Heinemann L, Lodwig V, Kurth HJ, Kolb H, et al Self-monitoring of blood glucose in type 2 diabetes and long-term outcome: an epidemiological cohort study. Diabetologia. 2006;49(2):271–8. doi: 10.1007/s00125-005-0083-5 16362814 10.1007/s00125-005-0083-5

[3] Vijan S, Stevens DL, Herman WH, Funnell MM, Standiford CJ. Screening, prevention, counseling, and treatment for the complications of type II diabetes mellitus. Putting evidence into practice. Journal of general internal medicine. 1997;12(9):567–80. doi: 10.1046/j.1525-1497.1997.07111.x 9294791 10.1046/j.1525-1497.1997.07111.x PMC1497162

[4] 6. Glycemic Targets. Diabetes care. 2015;38(Supplement 1):S33–S40. 25537705 10.2337/dc15-S009

[5] Morrison F, Shubina M, Turchin A. Encounter frequency and serum glucose level, blood pressure, and cholesterol level control in patients with diabetes mellitus. Archives of internal medicine. 2011;171(17):1542–50. doi: 10.1001/archinternmed.2011.400 21949161 10.1001/archinternmed.2011.400 PMC3692291

[6] Standards of Medical Care in Diabetes—2010. Diabetes care. 2010;33(Supplement 1):S11–S61. 20042772 10.2337/dc10-S011 PMC2797382

[7] Rodbard HW, Jellinger PS, Davidson JA, Einhorn D, Garber AJ, Grunberger G, et al Statement by an American Association of Clinical Endocrinologists/American College of Endocrinology consensus panel on type 2 diabetes mellitus: an algorithm for glycemic control. Endocrine practice: official journal of the American College of Endocrinology and the American Association of Clinical Endocrinologists. 2009;15(6):540–59. 10.4158/EP.15.6.540 19858063

[8] Kaufman FR, Halvorson M, Carpenter S. Association between diabetes control and visits to a multidisciplinary pediatric diabetes clinic. Pediatrics. 1999;103(5 Pt 1):948–51. 10224170 10.1542/peds.103.5.948

[9] Fenton JJ, Von Korff M, Lin EH, Ciechanowski P, Young BA. Quality of preventive care for diabetes: effects of visit frequency and competing demands. Annals of family medicine. 2006;4(1):32–9. doi: 10.1370/afm.421 16449394 10.1370/afm.421 PMC1466990

[10] Ashman JJ, Talwalkar A, Taylor SA. Age differences in visits to office-based physicians by patients with diabetes: United States, 2010. NCHS data brief. 2014(161):1–8. 25077512

[11] Munshi MN, Segal AR, Suhl E, Ryan C, Sternthal A, Giusti J, et al Assessment of barriers to improve diabetes management in older adults: a randomized controlled study. Diabetes care. 2013;36(3):543–9. doi: 10.2337/dc12-1303 23193208 10.2337/dc12-1303 PMC3579376

[12] Turchin A, Goldberg SI, Shubina M, Einbinder JS, Conlin PR. Encounter frequency and blood pressure in hypertensive patients with diabetes mellitus. Hypertension (Dallas, Tex: 1979). 2010;56(1):68–74. 10.1161/HYPERTENSIONAHA.109.148791 PMC3752696 20497991

[13] Aikens JE, Piette JD. Longitudinal association between medication adherence and glycaemic control in Type 2 diabetes. Diabetic medicine: a journal of the British Diabetic Association. 2013;30(3):338–44. 23075262 10.1111/dme.12046 PMC3567301

[14] Hoerger TJ, Segel JE, Gregg EW, Saaddine JB. Is glycemic control improving in U.S. adults? Diabetes care. 2008;31(1):81–6. doi: 10.2337/dc07-1572 17934153 10.2337/dc07-1572

[15] Takahashi PY, St Sauver JL, Finney Rutten LJ, Jacobson RM, Jacobson DJ, McGree ME, et al Health outcomes in diabetics measured with Minnesota Community Measurement quality metrics. Diabetes, metabolic syndrome and obesity: targets and therapy. 2015;8:1–8. 10.2147/DMSO.S71726 PMC4274142 25565873

[16] Ross SA. Breaking down patient and physician barriers to optimize glycemic control in type 2 diabetes. The American journal of medicine. 2013;126(9 Suppl 1):S38–48. doi: 10.1016/j.amjmed.2013.06.012 23953078 10.1016/j.amjmed.2013.06.012

[17] Age-Adjusted Percentage of Adults with Diabetes Using Diabetes Medication, by Type of Medication, United States, 1997–2011.

[18] Patel I, Chang J, Shenolikar RA, Balkrishnan R. Medication adherence in low income elderly type-2 diabetes patients: A retrospective cohort study. International Journal of Diabetes Mellitus. 2010;2(2):122–4.

[19] National Diabetes Fact Sheet: National Estimates and General Information on Diabetes and Prediabetes in the United States, 2011. 2011 [https://www.cdc.gov/diabetes/pubs/pdf/ndfs_2011.pdf.

[20] Ciechanowski PS, Katon WJ, Russo JE. Depression and diabetes: impact of depressive symptoms on adherence, function, and costs. Archives of internal medicine. 2000;160(21):3278–85. 11088090 10.1001/archinte.160.21.3278

[21] Gregg EW, Beckles GL, Williamson DF, Leveille SG, Langlois JA, Engelgau MM, et al Diabetes and physical disability among older U.S. adults. Diabetes care. 2000;23(9):1272–7. 10977018 10.2337/diacare.23.9.1272

[22] Huang ES, John P, Munshi MN. Multidisciplinary approach for the treatment of diabetes in the elderly. 2009.

[23] Bhattacharyya OK, Estey EA, Rasooly IR, Harris S, Zwarenstein M, Barnsley J. Providers' perceptions of barriers to the management of type 2 diabetes in remote Aboriginal settings. International journal of circumpolar health. 2011;70(5):552–63. 22067097 10.3402/ijch.v70i5.17848

[24] De Geest S, Sabate E. Adherence to long-term therapies: evidence for action. European journal of cardiovascular nursing: journal of the Working Group on Cardiovascular Nursing of the European Society of Cardiology. 2003;2(4):323. 10.1016/S1474-5151(03)00091-4 14667488

[25] Osterberg L, Blaschke T. Adherence to medication. The New England journal of medicine. 2005;353(5):487–97. doi: 10.1056/NEJMra050100 16079372 10.1056/NEJMra050100

